# Specialization in Plant-Hummingbird Networks Is Associated with Species Richness, Contemporary Precipitation and Quaternary Climate-Change Velocity

**DOI:** 10.1371/journal.pone.0025891

**Published:** 2011-10-05

**Authors:** Bo Dalsgaard, Else Magård, Jon Fjeldså, Ana M. Martín González, Carsten Rahbek, Jens M. Olesen, Jeff Ollerton, Ruben Alarcón, Andrea Cardoso Araujo, Peter A. Cotton, Carlos Lara, Caio Graco Machado, Ivan Sazima, Marlies Sazima, Allan Timmermann, Stella Watts, Brody Sandel, William J. Sutherland, Jens-Christian Svenning

**Affiliations:** 1 Conservation Science Group, Department of Zoology, University of Cambridge, Downing Street, Cambridge, United Kingdom; 2 Center for Macroecology, Evolution and Climate, Department of Biology, University of Copenhagen, Copenhagen, Denmark; 3 Department of Bioscience, Aarhus University, Aarhus, Denmark; 4 Center for Massive Data Algorithmics, Department of Computer Science, Aarhus University, Aarhus, Denmark; 5 Center for Macroecology, Evolution and Climate, Natural History Museum of Denmark, University of Copenhagen, Copenhagen, Denmark; 6 Pacific Ecoinformatics and Computational Ecology Lab, Berkeley, California, United States of America; 7 Landscape and Biodiversity Research Group, School of Science and Technology, University of Northampton, Northampton, United Kingdom; 8 Biology Program, California State University, Channel Islands, Camarillo, California, United States of America; 9 Departamento de Biologia, Universidade Federal de Mato Grosso do Sul, Campo Grande, Brazil; 10 Marine Biology and Ecology Research Centre, University of Plymouth, Plymouth, United Kingdom; 11 Centro Tlaxcala de Biología de la Conducta, Universidad Autónoma de Tlaxcala, Tlaxcala, México; 12 Laboratório de Ornitologia, Departamento de Ciências Biólogicas, Universidade Estadual de Feira de Santana, Feira de Santana, Bahia, Brazil; 13 Museu de Zoologia, IB, Universidade Estadual de Campinas, Campinas, Brazil; 14 Departamento de Biologia Vegetal, IB, Universidade Estadual de Campinas, Campinias, Brazil; 15 Laboratory of Pollination Ecology, Institute of Evolution, University of Haifa, Haifa, Israel; Institut Mediterrani d'Estudis Avançats (CSIC/UIB), Spain

## Abstract

Large-scale geographical patterns of biotic specialization and the underlying drivers are poorly understood, but it is widely believed that climate plays an important role in determining specialization. As climate-driven range dynamics should diminish local adaptations and favor generalization, one hypothesis is that contemporary biotic specialization is determined by the degree of past climatic instability, primarily Quaternary climate-change velocity. Other prominent hypotheses predict that either contemporary climate or species richness affect biotic specialization. To gain insight into geographical patterns of contemporary biotic specialization and its drivers, we use network analysis to determine the degree of specialization in plant-hummingbird mutualistic networks sampled at 31 localities, spanning a wide range of climate regimes across the Americas. We found greater biotic specialization at lower latitudes, with latitude explaining 20–22% of the spatial variation in plant-hummingbird specialization. Potential drivers of specialization - contemporary climate, Quaternary climate-change velocity, and species richness - had superior explanatory power, together explaining 53–64% of the variation in specialization. Notably, our data provides empirical evidence for the hypothesized roles of species richness, contemporary precipitation and Quaternary climate-change velocity as key predictors of biotic specialization, whereas contemporary temperature and seasonality seem unimportant in determining specialization. These results suggest that both ecological and evolutionary processes at Quaternary time scales can be important in driving large-scale geographical patterns of contemporary biotic specialization, at least for co-evolved systems such as plant-hummingbird networks.

## Introduction

Plant and animal assemblages do not live and evolve in isolation, but are entangled in networks of generalized and specialized biotic interactions [1]–[4]. Biotic specialization plays a central role in species coexistence and possible speciation [5], [6], and spatial variation in biotic specialization may therefore drive fundamental biodiversity patterns, such as the latitudinal species richness gradient [5]–[11]. Despite its importance, the underlying mechanisms that cause large-scale geographical differences in biotic specialization remain poorly understood [5]–[10]. Even the paradigm that biotic specialization is stronger in tropical than in sub-tropical and temperate assemblages is based on weak and contrasting quantitative evidence [6]–[9], [12]–[16].

Here we use mutualistic plant-hummingbird interaction networks to assess latitudinal patterns in contemporary specialization and, in order to move beyond the descriptive latitudinal specialization gradient, test whether contemporary specialization is most strongly associated with species richness, Quaternary climate-change velocity or contemporary climates [6]–[10], [17]–[21]. Mutualistic plant-hummingbird networks are ecologically important and well suited for such a large-scale comparative analysis. First of all, hummingbirds and their nectar plants are mutually dependent and biotic specialization plays an important role in structuring both local assemblages [19]–[27] and large-scale biodiversity patterns [11], [17], [21]. Second, hummingbirds and their nectar plants are relatively easy to observe and identify and studies of their interaction networks are therefore well resolved. In particular, studies typically report link strength between plants and hummingbirds, a surrogate for the mutualistic importance of an interaction [28]. Link strength is essential for a comparative analysis, as specialization indices computed from binary presence/absence networks - such as connectance [13], [14] - are sensitive to sampling effort and network size [29], [30].

Historical and evolutionary factors have been shown to affect species specialization level and the web of species with which species interact [31]–[33]. This suggests that contemporary mutualistic networks may be affected by their evolutionary history, and cannot be fully explained by contemporary ecological mechanisms [32], [33]. Extant hummingbirds radiated in the Early Miocene ∼17 Ma [17], giving ample time for long-term historical effects to accumulate in contemporary plant-hummingbird networks. However, contemporary plant-hummingbird assemblages, and their associated interaction networks, do not necessarily consist of species that have co-occurred and co-evolved over millions of years [17]. One factor that may have broken up species pairs is range-size dynamics associated with Quaternary climate fluctuations, which has long been considered important in shaping contemporary patterns of plant and animal diversity [6], [34]–[43]. Therefore, although plant-hummingbird associations *per se* have existed for millions of years - and age of plant-hummingbird associations may differ geographically (e.g., related to orogenic activity, such as the Andean uplift) [17] - climatic stability on Quaternary time scales may still capture important ecological and evolutionary processes in local plant-hummingbird networks. Traditionally, Quaternary climate change has been described as climatic anomaly (i.e., the change in mean climate at a given location), but it has recently been demonstrated that Quaternary climate-change velocity, incorporating both the climatic anomaly and topographic relief, and thus estimating the speed at which climates have moved across landscapes, is more biologically meaningful [43], [44]. Because Quaternary climate-change velocity combines information on both global patterns of climate change and local spatial gradients in climate, it provides a globally consistent description of climate instability that is scaled to local conditions. Furthermore, it captures the ability of topographic heterogeneity to buffer ecological communities from the effects of climate change. For example, a 1°C temperature increase has very different biological effects depending on the local topography. In mountainous areas, a short movement uphill would be sufficient to track a 1°C temperature increase, whereas relatively long distance movement would be needed in flat areas [43], [44]. Thus, we use climate-change velocity to describe climatic stability and test the hypothesis that climate-change velocity since the Late Quaternary (Last Glacial Maximum ∼21 ka) is negatively related to biotic specialization [6] in contemporary plant-hummingbird networks.

Contemporary climatic conditions provide a competing, or complementary, putative driver of contemporary plant-hummingbird specialization. Local and regional studies in South- and Central- America and the West Indies have addressed the role of contemporary climate on plant-hummingbird interactions [17], [19], [21], [26], [45]–[47]. These show that contemporary climates favorable for hummingbird-pollination are high precipitation [21], [45], [46], or the combination of high precipitation and relatively low temperatures [17], [19], [26], [47]. Such environments provide poor flying conditions for insects, resulting in inefficient insect-pollination [19], [26], [47]. Hummingbirds are physiologically less affected by environmental conditions than most insect-pollinators, which may lead to greater interdependence and specialization between plants and hummingbirds in areas of high precipitation and low temperatures [17], [19], [26], [45]–[47]. However, theory also suggests that areas of high productivity may offer greater opportunities for specialization [7], [10], and therefore predicts that both precipitation and temperature should be positively related to biotic specialization. A recent analysis of the phylogenetic structure of hummingbird assemblages along environmental gradients in the Ecuadorian Andes also indicated that biotic interactions may play a noticeable role in structuring hummingbird assemblages in the humid lowlands, whereas ecological filtering appeared to be relatively more important in the cool highlands [20]. Hence, studies of pollination ecology, productivity and hummingbird phylogenetic structure all predict that precipitation should be positively related to specialization, whereas they differ on the role of temperature. Furthermore, contemporary seasonality may also affect specialization [18], [21]. In areas with low precipitation seasonality or temperature seasonality, resources may be more constant and plant-hummingbird assemblages may therefore show increased specialization to these local conditions [18], [21]. In addition to Quaternary climate-change velocity and contemporary climate, a long-standing tenet in evolutionary ecological theory is the positive relationship between species richness and biotic specialization, i.e., that large plant-hummingbird assemblages are more specialized than small assemblages due to finer division of resources [5], [13], [15], [48].

**Figure 1:**
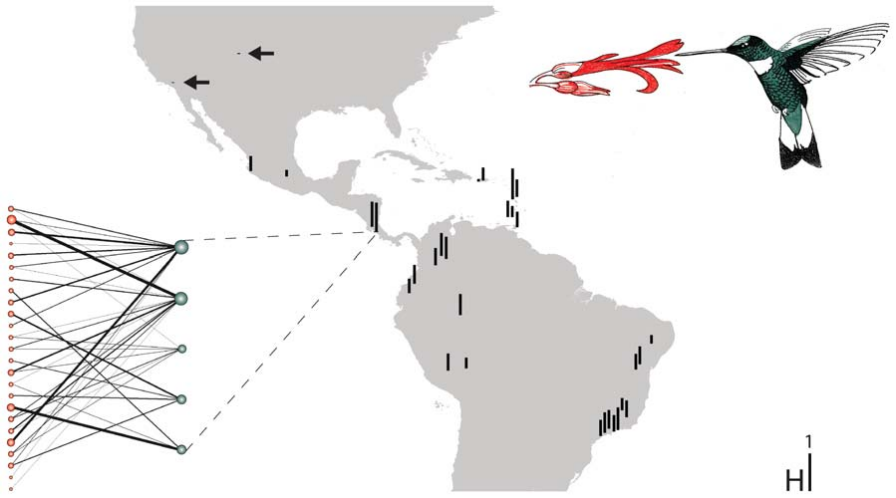
Geographical patterns of contemporary plant-hummingbird specialization. Map of the Americas showing degree of specialization (H) in native plant-hummingbird networks. Arrows indicate studies that are difficult to see due to low specialization. The network to the left depicts an extremely specialized network (H = 0.78, P<0.05) from the Costa Rican highlands at latitude 9°N. The red nodes to the left illustrate plant species, and the green nodes to the right hummingbird species. The widths of links are scaled to interaction frequency, and node sizes to total interaction frequency. It illustrates how low Quaternary climate-change velocity, high contemporary precipitation and high species richness may cause strong contemporary biotic specialization. See Table S1 for specialization estimates for networks containing both native and introduced species.

To gain insight into geographical patterns of contemporary plant-hummingbird specialization and its drivers, we compiled 31 quantitative plant-hummingbird interaction networks, spanning a wide range of elevation and climate regimes across the Americas (Figure 1; Table S1). First, we tested whether each network was more specialized than expected at random. We then examined whether specialization in plant-hummingbird networks was negatively correlated with latitude, i.e., whether tropical plant-hummingbird assemblages are more specialized than sub-tropical and temperate assemblages. Finally, we tested whether network size, contemporary climate and/or climate-change velocity since the Quaternary determines contemporary plant-hummingbird specialization. As introduced species may distort potential relationships between specialization and latitude, network size, contemporary climate and Quaternary climate-change velocity, we conducted the entire analysis twice: once just for native plant-hummingbird networks, excluding introduced species, and once for networks where introduced plant species were included.

## Results

For each of the 31 plant-hummingbird networks, we measured network-level contemporary specialization (H), and assessed whether the level of specialization was higher than expected at random [29]. Irrespective of whether introduced plant species were included or excluded, specialization was higher than expected in all except the smallest plant-hummingbird networks (P<0.05; Table S1).

### Latitudinal patterns of specialization

**Figure 2:**
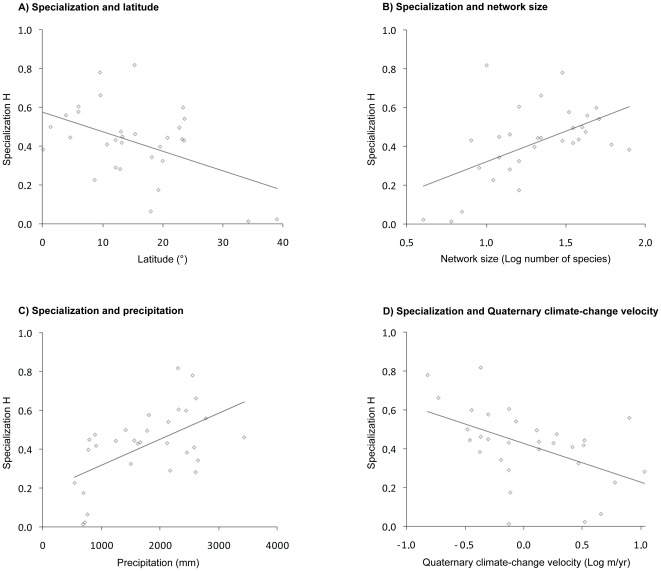
Relationship of contemporary specialization with latitude and underlying drivers. (A) Linear relationship between latitude and specialization in plant-hummingbird networks (H: n = 31, R^2^ = 0.22, Dutilleul's spatially corrected P<0.05). (B) Linear relationship between Log-transformed network size and specialization in plant-hummingbird networks (H: n = 31, R^2^ = 0.28, Dutilleul's spatially corrected P<0.01). (C) Linear relationship between mean annual precipitation and specialization in plant-hummingbird networks (H: n = 31, R^2^ = 0.31, Dutilleul's spatially corrected P<0.01). (D) Linear relationship between Log-transformed Quaternary climate-change velocity and specialization in plant-hummingbird networks (H: n = 31, R^2^ = 0.25, Dutilleul's spatially corrected P<0.05). Each symbol represents a native plant-hummingbird network. See Tables 1 and S2 for predictor estimates in ordinary-least-squares (OLS) multiple regression models. Likewise, see Tables 1 and S3 for predictor estimates in plant-hummingbird networks containing both native and introduced species.

Spatially, we first examined how contemporary specialization correlates with latitude. We corrected significance level for spatial autocorrelation, using Dutilleul's method [49]. Specialization was significantly negatively related to latitude both for native plant-hummingbird networks (Figure 2; H: n = 31, R^2^ = 0.22, Dutilleul's P<0.05) and when introduced plants were included (H: n = 31, R^2^ = 0.20, Dutilleul's P<0.05).

### Determinants of specialization

In order to determine what may cause low/high contemporary plant-hummingbird specialization, for the geographical locality of each network we obtained estimates of climate-change velocity since Last Glacial Maximum (VELOCITY), and four variables describing the contemporary climate: mean annual temperature (MAT), mean annual precipitation (MAP), temperature seasonality (SEAS_T_), and precipitation seasonality (SEAS_P_). In addition, we included the species richness of the plant-hummingbird network (SIZE) and the length of the study period (DAYS) as observed specialization may be affected by seasonal phenological displacement ([50]; Materials and Methods; Table S1).

We then determined the role of Quaternary climate-change velocity and contemporary climate as determinants of contemporary specialization, taking into account network size and length of the study period. We did this by comparing several ordinary-least-squares (OLS) multiple regression models grouped into three types: 1) a “velocity” model, with VELOCITY as predictor of specialization; 2) “contemporary climate” models, with various likely combinations of MAP, MAT, SEAS_T_, and SEAT_P_ as predictors; and 3) “velocity and contemporary climate” models, incorporating likely combinations of VELOCITY, MAP, MAT, SEAS_T_, and SEAT_P_ into the same models (Materials and Methods; Tables S2, S3). In all models, we also included network size (SIZE) and length of study period (DAYS), controlling for these potentially confounding factors. Our main analysis focused on plant-hummingbird networks containing only native species (Figures 1–2; Table S2). In addition, we checked the sensitivity of the obtained results when including introduced species (Table S3). Based on the initially identified best-fit models (Tables S2, S3), we subsequently used an Akaike information criterion (AIC_c_) forward-selection procedure to reduce the number of predictors until all predictors in the best-fit models were significant (i.e., P≤0.05), forming the basis of our discussion (Table 1). It was not necessary to correct for spatial autocorrelation in any of our multiple regression models, as the residuals in no case exhibited significant positive spatial autocorrelation (Tables 1 and S2, S3). See Materials and Methods for a detailed description of the analytical approach.

**Table 1:** Multiple regression models predicting contemporary specialization in plant-hummingbird networks.

Origin	SIZE	MAP	VELOCITY	R^2^_adj_	Moran's I	VIF	CN
Native species	+0.38**	+0.34*	−0.34*	0.53**	I≤0.13^NS^	≤1.2	1.6
Native and introduced species	+0.50**	+0.29*	−0.33*	0.64**	I≤0.16^NS^	≤1.2	1.6

Predictor estimates are for each model given as standardized regression coefficients. Predictors included in the best-fit multiple regression models are: network size, i.e, species richness in the network (SIZE); mean annual precipitation (MAP); Quaternary climate-change velocity (VELOCITY). None of the other predictors included in the analysis - length of study period (DAYS); mean annual temperature (MAT); precipitation seasonality (SEAS_P_); temperature seasonality (SEAS_T_) - were included in any of the best-fit models, and are therefore not included here. Moran's I and VIF/CN show that neither positive spatial autocorrelation nor multicollinearity was a problem in our models. See Tables S2, S3 and Materials and Methods for modelling approach.

**P<0.01,

*P<0.05,

NS P>0.05.

In the best-fit models for native plant-hummingbird networks, contemporary specialization (H) was positively related to network size and mean annual precipitation, and negatively to Quaternary climate-change velocity (Figure 2; Table 1). For networks including introduced plant species, we obtained similar results to those containing native species only (Table 1). Neither mean annual temperature, seasonality nor the length of the study period were included in any of the best-fit models. Across all analyses, network size was the most important predictor of specialization, followed by Quaternary climate-change velocity and contemporary mean annual precipitation (Table 1).

## Discussion

While the majority of mutualistic plant-pollinator interactions are believed to be moderately generalized [13], [16], [51], we show that plant-hummingbird mutualistic networks are more specialized than expected at random (Table S1). With respect to latitude, our data confirm that tropical plant-hummingbird networks are more specialized than sub-tropical and temperate networks. However, latitude only explained a maximum of 22% of the spatial variation in specialization. This is consistent with the weak and mixed results of previous studies evaluating the latitudinal specialization gradient in plant-pollinator assemblages [8], [12]–[16], and echoes the call of some biogeographers for a more mechanistic approach, seeking to understand the underlying environmental drivers - many of which are components of climate [52]. In accordance with this, we show that contemporary climate and Quaternary climate-change velocity together with species richness performed much better than latitude as predictors of specialization, explaining up to 64% of the variation in plant-hummingbird specialization.

Our results agree with previous local and regional studies in South- and Central- America and the West Indies that contemporary climates that provide poor conditions for insect-pollination (high precipitation) lead to greater interdependence and specialization between plants and hummingbirds (Tables 1 and S2, S3; [17], [19], [21], [26], [45]–[47]). Besides contemporary climates, we show that strong biotic specialization is tightly linked to species-rich networks and low Quaternary climate-change velocity (Tables 1 and S2, S3). Although the exact mechanism behind the link between contemporary specialization and Quaternary climate-change velocity cannot be determined by the present study, our findings support the hypothesis that low Quaternary climate-change velocity - and the associated persistence of species - increases local adaptation and favor specialization in biotic systems [6]. These results have significant impact on ecological and evolutionary theory predicting geographic patterns of contemporary biotic specialization [6], and may also help understand why patterns of biodiversity are associated with Quaternary climate-change velocity [6], [34]–[43].

In regard to climate-change, our study shows that it may be crucial to include Quaternary climate-change velocity as a predictor of contemporary biotic specialization, also when evaluating the effect of contemporary climate on mutualistic systems – an important and recurrent exercise these days [30], [53]–[61]. In a global context, our results predict that biotic specialization and co-evolution should be especially strong in mountainous biomes (e.g., in the Andes and Central American mountains as observed for plant-hummingbird networks) and other areas with low Quaternary climate-change velocity, whereas flatter landscapes particularly at high latitudes should consist of mainly generalized mutualistic networks. However, it remains to be tested whether the strong signal of Quaternary climate-change velocity observed in plant-hummingbird networks across the Americas can be extrapolated to a broad range of mutualistic systems across the globe.

## Materials and Methods

### Plant-hummingbird networks

We compiled all published studies that have recorded plant-hummingbird interactions for entire plant-hummingbird assemblages, as well as our own unpublished plant-hummingbird interaction networks. We considered only mutualistic interactions, excluding interactions in which hummingbirds acted as nectar robbers without pollinating the plant. In order to be included in the analysis, the plant-hummingbird networks had to fulfill three quality criteria: 1) the link strength of each plant-hummingbird interaction had to be reported, i.e., we discarded all binary datasets considering only whether an interaction occurred or not. We did this because specialization indices computed from binary presence/absence networks are sensitive to sampling effort and network size - making cross-network comparisons based on binary networks unreliable [29], [30]; 2) the link strength had to be based on visitation rate, i.e., we did not include datasets measuring interaction strength based solely on pollen load analysis; 3) the assemblage had to contain at least two plant and two hummingbird species. Hence, studies from southern Chile and Argentina, where only one hummingbird species exists, were not included in the analysis. Furthermore, one network was discarded from the analysis as it had been sampled in a university campus and contained 64% introduced plant species [62].

Of the networks included in the analysis, 14 contained on average 11% introduced plant species. All the remaining networks only contained native species. In order to assess the sensitivity of the results to introduced species, we created two datasets - one only including native plant species, and another one including both native and introduced species. We checked plant origin using the information provided in the original publication, if mentioned, combined with various web resources, principally Tropicos (www.tropicos.org), Grin Taxonomy for Plants (www.ars-grin.gov), and Flora of West Indies at the Smithsonian National Museum of Natural History (www.botany.si.edu/Antilles/WestIndies), as well as other literature sources. In those cases where a plant was only identified to genus level, it was included as a native species if the genus is found naturally in the given locality; otherwise it was coded as an introduced species. In total, we were able to obtain 31 high-quality, quantitative plant-hummingbird networks (Table S1).

### Predictor variables

For each of the 31 study localities we obtained the geographical position (latitude and longitude) and corresponding estimates of potential Quaternary and contemporary climate drivers of specialization (Table S1). As a Quaternary climate predictor, we estimated climate-change velocity (m/yr) since Last Glacial Maximum (VELOCITY). Climate-change velocity describes the rate at which climate conditions are moving over the Earth's surface at any particular point [43], [44]. It is calculated by dividing a temporal climate gradient (e.g., C/yr) by a spatial gradient (e.g., C/km) [43], [44]; in this case our temporal gradient was the change in mean annual temperature (MAT) for each grid cell since the Last Glacial Maximum (LGM), while the spatial gradient was the local slope of the current MAT surface. The slope of the MAT surface at a particular grid cell was calculated using the average maximum technique, accounting for latitudinal variation in cell size. Using this technique, the slope value for a cell is determined by the relative MAT values of the neighboring cells. Velocities were calculated at 2.5 minute grid cell resolution (approximately 21.4 km^2^ at the equator) and then aggregated to a global 0.25 degree resolution map (approximately 770 km^2^ at the equator). We calculated velocity at this fairly fine scale to capture potentially important effects of small-scale topoclimate gradients [43], [44]. Paleoclimate data were obtained from the Paleoclimate Modeling and Intercomparison Project Phase II (PMIP-2; [63]) CCSM3 and MIROC 3.2 models. We used the average of these two model predictions as our estimate of LGM MAT. Because climate-change velocity combines information on both global patterns of climate change and local spatial gradients in climate, it provides a globally consistent description of climate instability that is scaled to local conditions. Furthermore, it captures, quantitatively, the ability of topographic heterogeneity to buffer ecological communities from the effects of climate change. As contemporary climate predictors we included mean annual temperature (C*10; MAT), mean annual precipitation (mm; MAP), temperature seasonality (standard deviation *100; SEAS_T_) and precipitation seasonality (coefficient of variation; SEAS_P_). All contemporary climate data were extracted with a 30 second resolution from WorldClim 1.4 ([64]; http://www.worldclim.org/). In addition, we included the species richness of the interacting plant-hummingbird assemblage (SIZE), i.e., the number of plant and hummingbird species. Furthermore, due to seasonal phenological displacement [50], observed specialization may be related to the length of the study period (1–365 days), which was therefore also included (DAYS). For correlations between predictor variables, see Table S4.

### Data analysis

We conducted the analysis for native plant-hummingbird networks, excluding introduced species, but also repeated the entire analysis when including introduced species. We emphasise our results obtained for native plant-hummingbird networks more than those obtained when introduced species were included. We do this because only interactions between native species reflect co-evolved associations potentially affected by historical factors, and because some studies excluded introduced species. Hence, by focusing on native plant-hummingbird networks, we ensure both a co-evolutionary history between assemblages of hummingbirds and their nectar plants and, equally importantly, we are not introducing sampling bias between networks.

For each of the 31 quantitative plant-hummingbird networks (Table S1), we measured network-level contemporary specialization (H), using the method and software of Blüthgen and co-workers ([29]; http://rxc.sys-bio.net/). The degree of specialization was measured as two-dimensional Shannon entropy and standardized to range between 0 and 1 for extreme generalization and specialization, respectively (for equations, see [29]). We used a null-model to assess whether specialization level was higher than expected at random. In the null model, each species was assigned the same total number of interactions as in the sampled matrix, but interactions were assigned at random. The probability that the sampled network had a higher specialization level than expected by random (i.e., the significance level) was calculated as the proportion of values obtained by random (10,000 permutations) that were equal or larger than the specialization value for the sampled network. For more information about the methods, see the work by Blüthgen and co-workers ([29]; http://rxc.sys-bio.net/).

We then correlated specialization with absolute latitude. The significance level was calculated with the degrees of freedom and significance level corrected for spatial autocorrelation, using Dutilleul's method [49]. We thereafter examined how Quaternary climate-change velocity and contemporary climate relate to specialization, taking into account network size and the length of the study period. For this we compared seven ordinary-least-squares (OLS) multiple regression models grouped into three types: 1) a “velocity” model, with VELOCITY as predictor of specialization; 2) three “contemporary climate” models increasing in complexity. The simplest model only included MAP, following pollination ecology studies that show that precipitation may affect specialization [21], [45], [46]. The second model included MAP and MAT, following those studies that suggest that both precipitation and temperature may affect plant-hummingbird specialization [17], [19], [26], [47]. The most complex contemporary climate model further included seasonality, i.e., variables MAT, MAP, SEAS_T_, and SEAT_P_ as predictors; and 3) three combined “velocity and contemporary climate” models, increasing in complexity as the contemporary climate models (e.g., Table S2). All models also included network size (SIZE) and length of study period (DAYS), controlling for these potentially confounding factors. Subsequently, based on the initially identified best-fit models (Tables S2-S3) we used an Akaike information criterion (AIC_c_) forward-selection procedure to reduce the number of predictors until all predictors were significant (i.e., P≤0.05; Table 1). The variables SIZE, VELOCITY and SEAS_T_ were log_10_ transformed as this improved the assumptions of linearity and diminished potential problems with outliers. We evaluated the likelihood of each of the models using the Akaike information criterion AIC_c_ and R^2^_adj_. We assessed whether significant positive spatial autocorrelation remained in the models based on Moran's I with eight distance classes and a permutation test (with 10,000 iterations) on the residual spatial autocorrelation. Finally, we checked for multicollinearity using the condition number (CN) and the variance inflation factor (VIF). Neither positive spatial autocorrelation nor multicollinearity was a problem in our models (Tables 1 and S2-S3). Hence, we did not build more complicated models. The software Spatial Analysis in Macroecology SAM 4.0 ([65]; http://www.ecoevol.ufg.br/sam/) was used for multiple regression and spatial analysis tests.

## Supporting Information

Table S1 **Plant-hummingbird networks: response and predictor variables.**
(DOC)

Table S2 **Models predicting contemporary specialization in native plant-hummingbird networks.**
(DOC)

Table S3 **Models predicting contemporary specialization in plant-hummingbird networks including introduced species.**
(DOC)

Table S4 **Correlations between predictor variables.**
(DOC)
