# Supplementary material for: Specialization in Plant-Hummingbird Networks Is Associated with Species Richness, Contemporary Precipitation and Quaternary Climate-Change Velocity
Source: PLoS One. 2011 Oct 5;6(10):e25891. doi: 10.1371/journal.pone.0025891 (PMC3187835; doi:10.1371/journal.pone.0025891)
Supplement: Table S1 — Plant-hummingbird networks: response and predictor variables. (DOC) [file pone.0025891.s001.doc]

**Table S1. Plant-hummingbird networks: response and predictor variables.** In each of the 31 plant-hummingbird networks, we measured network-level specialization (H). In order to assess the sensitivity of results to introduced species, we created and measured specialization in two datasets - one dataset only including native plant species (“native”), and another one including both native and introduced species (“native + introduced”). Predictors of specialization are: network size, i.e., species richness in the network (SIZE), calculated both for “native” networks and “native + introduced” networks. The remaining predictor variables were calculated for each specific location. These are: length of study period, 1-365 days (DAYS); mean annual precipitation (MAP); mean annual temperature (MAT); precipitation seasonality (SEAS_P_); temperature seasonality (SEAS_T_); and climate-change velocity since Last Glacial Maximum (VELOCITY). See Materials and Methods for details of how each predictor variable was calculated. The studies are arranged from North to South.

|  |  | Response variables | |  | Predictor variables | | | | | | | |
| --- | --- | --- | --- | --- | --- | --- | --- | --- | --- | --- | --- | --- |
|  |  | native | native + introduced |  | native | native + introduced | locality specific | | | | | |
| Source | Geographic coordinates | H^†^ | H^†^ |  | SIZE | SIZE | DAYS | MAP | MAT | SEAS_P_ | SEAS_T_ | VELOCITY |
| 1 | 38°59´N, 106°58´W | 0.02^NS^ | 0.02^NS^ |  | 4 | 4 | 39 | 721 | -23 | 16 | 7718 | 3.33 |
| 2 | 34°13´N, 116°57´W | 0.01^NS^ | 0.01^NS^ |  | 6 | 6 | 42 | 691 | 71 | 72 | 5880 | 0.75 |
| 3 | 19°30´N, 105°03´W | 0.40^*^ | 0.40^*^ |  | 20 | 20 | 365 | 782 | 262 | 109 | 1651 | 1.35 |
| 4 | 19°14´N, 98°58´W | 0.17^*^ | 0.17^*^ |  | 16 | 16 | 365 | 699 | 158 | 90 | 1932 | 0.77 |
| 5 | 18°08´N, 66°46´W | 0.34^*^ | 0.34^*^ |  | 12 | 13 | 105 | 2647 | 196 | 47 | 1273 | 0.64 |
| 5 | 17°57´N, 66°49´W | 0.06^NS^ | 0.06^NS^ |  | 7 | 7 | 105 | 767 | 264 | 57 | 1127 | 4.58 |
| 5 | 15°21´N, 61°18´W | 0.46^*^ | 0.67^*^ |  | 14 | 15 | 99 | 3434 | 216 | 27 | 978 | 0.43 |
| 5 | 15°15´N, 61°22´W | 0.82^*^ | 0.47^*^ |  | 10 | 13 | 99 | 2305 | 252 | 39 | 993 | 0.43 |
| 5 | 12°06´N, 61°42´W | 0.43^*^ | 0.14^*^ |  | 8 | 9 | 108 | 2123 | 252 | 39 | 636 | 0.75 |
| 5 | 12°06´N, 61°41´W | 0.29^*^ | 0.37^*^ |  | 9 | 10 | 108 | 2174 | 249 | 39 | 634 | 0.75 |
| 6 | 10°40´N, 61°17´W | 0.41^*^ | 0.42^*^ |  | 61 | 66 | 365 | 2582 | 244 | 44 | 643 | 2.62 |
| 7 | 9°34´N, 83°44´W | 0.66^*^ | 0.66^*^ |  | 22 | 22 | 198 | 2613 | 91 | 67 | 609 | 0.19 |
| 7 | 9°29´N, 83°29´W | 0.78^*^ | 0.78^*^ |  | 30 | 30 | 9 | 2556 | 60 | 60 | 661 | 0.15 |
| 8 | 5°55´N, 73°32´W | 0.61^*^ | 0.51^*^ |  | 16 | 19 | 29 | 2316 | 195 | 39 | 256 | 0.75 |
| 8 | 5°54´N, 73°25´W | 0.58^*^ | 0.58^*^ |  | 33 | 34 | 25 | 1813 | 145 | 46 | 388 | 0.50 |
| 8 | 4°32´N, 73°51´W | 0.44^*^ | 0.44^*^ |  | 22 | 22 | 23 | 1563 | 148 | 40 | 337 | 0.35 |
| 9, 10 | 1°15´N, 77°26´W | 0.50^*^ | 0.50^*^ |  | 40 | 40 | 365 | 1417 | 131 | 44 | 243 | 0.33 |
| 11 | 0°01´S, 78°46´W | 0.38^*^ | 0.38^*^ |  | 79 | 84 | 70 | 2459 | 174 | 54 | 201 | 0.42 |
| 12 | 3°49´S, 70°16´W | 0.56^*^ | 0.54^*^ |  | 43 | 44 | 365 | 2780 | 260 | 22 | 403 | 8.00 |
| 13 | 8°36´S, 38°34´W | 0.23^*^ | 0.23^*^ |  | 11 | 11 | 365 | 548 | 255 | 88 | 1519 | 6.03 |
| 14 | 12°51´S, 69°22´W | 0.28^*^ | 0.25^*^ |  | 14 | 15 | 15 | 2608 | 253 | 51 | 1071 | 10.77 |
| 15 | 12°59´S, 41°20´W | 0.48^*^ | 0.48^*^ |  | 42 | 42 | 365 | 895 | 195 | 55 | 1252 | 1.92 |
| 16 | 13°07´S, 41°35´W | 0.42^*^ | 0.42^*^ |  | 35 | 35 | 365 | 915 | 196 | 55 | 1195 | 3.26 |
| 17 | 13°13´S, 72°07´W | 0.45^*^ | 0.45^*^ |  | 12 | 12 | 1 | 798 | 40 | 77 | 1397 | 0.50 |
| 18 | 19°57´S, 43°54´W | 0.32^*^ | 0.32^*^ |  | 16 | 16 | 365 | 1504 | 189 | 86 | 1812 | 2.95 |
| 19 | 20°45´S, 42°55´W | 0.44^*^ | 0.43^*^ |  | 21 | 22 | 365 | 1248 | 203 | 75 | 2178 | 3.31 |
| 20, 21 | 22°44´S, 45°35´W | 0.50^*^ | 0.50^*^ |  | 35 | 35 | 365 | 1782 | 136 | 67 | 2305 | 1.29 |
| 21 | 23°10´S, 44°55´W | 0.44^*^ | 0.44^*^ |  | 38 | 38 | 365 | 1666 | 153 | 64 | 2177 | 1.35 |
| 22 | 23°21´S, 44°51´W | 0.60^*^ | 0.60^*^ |  | 49 | 53 | 365 | 2445 | 232 | 39 | 2211 | 0.36 |
| 21 | 23°35´S, 45°20´W | 0.54^*^ | 0.54^*^ | | 51 | 51 | 365 | 2143 | 234 | 42 | 2271 | 0.86 |
| 23 | 23°38´S, 45°51´W | 0.43^*^ | 0.41^*^ |  | 30 | 31 | 37 | 1622 | 174 | 50 | 2285 | 1.80 |

^†^Significance level: ^*^<0.05, ^NS^P>0.05.

**Supplementary References**

1. Alarcón R (2004) The structure of plant-pollinator interactions in montane meadow environments: Ph.D. Thesis, University of California Riverside.
2. Alarcón R, Waser NM, Ollerton J (2008) Year-to-year variation in the topology of a plant-pollinator interaction network. Oikos 117: 1796-1807.
3. Arizmendi MC, Ornelas JF (1990) Hummingbirds and their floral resources in a tropical dry forest in Mexico. Biotropica 22: 172-180.
4. Lara C (2006) Temporal dynamics of flower use by hummingbirds in a highland temperate forest in Mexico. Ecoscience 13: 23-29.
5. Dalsgaard B, Martín Gonzáles AM, Olesen JM, Ollerton J, Timmermann A, et al. (2009) Plant-hummingbird interactions in the West Indies: floral specialisation gradients associated with environment and hummingbird size. Oecologia 159: 757-766.
6. Snow BK, Snow DW (1972) Feeding niches of hummingbirds in a Trinidad valley. J Anim Ecol 41: 471-485.
7. Wolf LL, Stiles GF, Hainsworth FR (1976) Ecological organization of a tropical, highland hummingbird community. J Anim Ecol 45: 349-379.
8. Snow DW, Snow BK (1980) Relationships between hummingbirds and flowers in the Andes of Colombia. Bull Br Mus Nat Hist (Zool) 38: 105-139.
9. Gutierres Aquiles EZ, Rojas-Nossa SV (2001) Dinámica anual de la interacción de colibrí-flor en ecosistemas altoandinos del volcán Galeras, sur de Colombia: Título de biología, Universidad Nacional de Colombia.
10. Gutierres Aquiles EZ, Rojas-Nossa SV, Stiles GF (2004) Dinámica anual de la interacción de colibrí-flor en ecosistemas altoandinos. Ornitologia Neotropical 15 (suppl.): 1-9.
11. Walther BA, Brieschke H (2001) Hummingbird-flower relationships in a mid-elevation rainforest near Mindo, northwestern Ecuador. Internat J Ornithol 4: 115-135.
12. Cotton PA (1998) Coevolution in an Amazonian hummingbird-plant community. Ibis 140: 639-646.
13. Leal FC, Lopes AV, Machado IC (2006) Polinização por beija-flores em uma área de caatinga no Município de Floresta, Pernambuco, nordeste do Brasil. Rev Bras Bot 29: 379-389.
14. Watts S (1998) Patterns of floral visitation and floral characteristics in relation to the morphology of visiting hummingbirds in southeastern Perú: B.Sc. Thesis, University of Northampton.
15. Machado CG, Coelho AG, Santana CS, Rodrigues M (2007) Beija-flores e seus recursos florais em uma area de campo rupestre da Chapada Diamantina, Bahia. Rev Bras Orn 15: 267-279.
16. Machado CG (2009) Beija-flores (Trochilidae) e seus recursos florais em uma area de caatinga da Chapada Diamantina, Bahia. Zoologia 26: 255-265.
17. Watts S (2008) Plant-flower visitor interactions in the Sacred Valley of Perú: Ph.D. Thesis, University of Northampton.
18. Vasconcelos MF, Lombardi JA (1999) Padrão sazonal na ocorrência de seis espécies de beija-flores (Apodiformes: Trochilidae) em uma localidade de campo rupestre na Serra do Curral, Minas Gerais. Ararajuba 7: 71-79.
19. Abreu CRM, Vieira MF (2004) Os beija-flores e seus recursos florais em um fragmento florestal de Viçosa, sudeste brasileiro. Lundiana 5: 129-134.
20. Sazima I, Buzato S, Sazima M (1996) An assemblage of hummingbird-pollinated flowers in a montane forest in southern Brasil. Bot Acta 109: 149-160.
21. Buzato S, Sazima M, Sazima I (2000) Hummingbird-pollinated floras at three Atlantic forest sites. Biotropica 32: 824-841.
22. Araujo AC (1996) Beija-flores e seus recursos florais numa área de planicie costeira do litoral norte de São Paulo: M.Sc. Thesis, Universidade Estadual de Campinas (UNICAMP).
23. Snow DW, Snow BK (1986) Feeding ecology of hummingbirds in the Serra Do Mar, southeastern Brazil. El Hornero 12: 286-296.
