# Supplementary material for: Specialization in Plant-Hummingbird Networks Is Associated with Species Richness, Contemporary Precipitation and Quaternary Climate-Change Velocity
Source: PLoS One. 2011 Oct 5;6(10):e25891. doi: 10.1371/journal.pone.0025891 (PMC3187835; doi:10.1371/journal.pone.0025891)
Supplement: Table S2 — Models predicting contemporary specialization in native plant-hummingbird networks. (DOC) [file pone.0025891.s002.doc]

**Table S2. Models predicting contemporary specialization in native plant-hummingbird networks**. Predictor estimates are for each model given as standardized regression coefficients. Predictors are: length of study period, 1-365 days (DAYS); network size, i.e., species richness in the network (SIZE); mean annual precipitation (MAP); mean annual temperature (MAT); precipitation seasonality (SEAS_P_); temperature seasonality (SEAS_T_); and climate-change velocity since Last Glacial Maximum (VELOCITY). In all models we included network size (SIZE) and length of study season (DAYS), controlling for the potentially confounding effects of these variables. Predictors not included in a given model are marked by a dash (―). Moran’s I and VIF/CN show that neither positive spatial autocorrelation nor multicollinearity was a problem in our models. The best-fit model is marked in **bold**.

|  | **Model group** | **DAYS** | **SIZE** | **MAP** | **MAT** | **SEAS_P_** | **SEAS_T_** | **VELOCITY** | **ΔAIC_c_** | **R^2^_adj_** | **Moran’s I** | **VIF** | **CN** |
| --- | --- | --- | --- | --- | --- | --- | --- | --- | --- | --- | --- | --- | --- |
|  | *velocity* | -0.05^NS^ | +0.50^**^ | ― | ― | ― | ― | -0.41^*^ | 3.06 | 0.43^**^ | I ≤ 0.16^NS^ | ≤ 1.6 | 2.0 |
|  | *contemporary climate* | -0.07^NS^ | +0.44^*^ | +0.41^*^ | ― | ― | ― | ― | 3.63 | 0.42^**^ | I ≤ 0.06^NS^ | ≤ 1.6 | 2.1 |
|  |  | -0.03^NS^ | +0.42^*^ | +0.44^*^ | -0.08^NS^ | ― | ― | ― | 6.49 | 0.40^**^ | I ≤ 0.06^NS^ | ≤ 1.9 | 2.6 |
|  |  | +0.05^NS^ | +0.30^NS^ | +0.48^*^ | -0.14^NS^ | +0.11^NS^ | -0.21^NS^ | ― | 11.58 | 0.39^**^ | I ≤ 0.11^NS^ | ≤ 2.4 | 3.3 |
|  | *velocity & contemporary climate* | **+0.08^NS^** | **+0.33^NS^** | **+0.36^*^** | **―** | **―** | **―** | **-0.37^*^** | **0** | **0.52^**^** | **I ≤ 0.10^NS^** | **≤ 1.7** | **2.2** |
|  |  | +0.04^NS^ | +0.34^NS^ | +0.31^NS^ | +0.10^NS^ | ― | ― | -0.40^*^ | 2.95 | 0.50^**^ | I ≤ 0.11^NS^ | ≤ 2.0 | 2.7 |
|  |  | +0.09^NS^ | +0.30^NS^ | +0.32^NS^ | +0.05^NS^ | +0.01^NS^ | -0.10^NS^ | -0.38^*^ | 10.26 | 0.47^**^ | I ≤ 0.14^NS^ | ≤ 2.4 | 3.6 |

^**^P<0.01, ^*^P<0.05, ^NS^P>0.05.
