# Supplementary material for: Specialization in Plant-Hummingbird Networks Is Associated with Species Richness, Contemporary Precipitation and Quaternary Climate-Change Velocity
Source: PLoS One. 2011 Oct 5;6(10):e25891. doi: 10.1371/journal.pone.0025891 (PMC3187835; doi:10.1371/journal.pone.0025891)
Supplement: Table S4 — Correlations between predictor variables. (DOC) [file pone.0025891.s004.doc]

**Table S4. Correlations between predictor variables.** Significance level is corrected for spatial autocorrelation, using Dutilleul’s method [49]. Notice that most variables are non-significantly correlated, and multicollinearity is not a problem in any of our multiple regression models (Table 1; Tables S2-S3). See Table S1 or Materials and Methods for a description of each variable.

|  | **DAYS** | **SIZE** | **MAP** | **MAT** | **SEAS_P_** | **SEAS_T_** | **VELOCITY** |
| --- | --- | --- | --- | --- | --- | --- | --- |
| **DAYS** | ― |  |  |  |  |  |  |
| **SIZE** | +0.46^*^ | ― |  |  |  |  |  |
| **MAP** | -0.19^NS^ | +0.31^NS^ | ― |  |  |  |  |
| **MAT** | +0.35^NS^ | +0.16^NS^ | +0.27^NS^ | ― |  |  |  |
| **SEAS_P_** | +0.34^NS^ | -0.03^NS^ | -0.55^*^ | -0.02^NS^ | ― |  |  |
| **SEAS_T_** | +0.18^NS^ | -0.38^NS^ | -0.47^NS^ | -0.25^NS^ | +0.27^NS^ | ― |  |
| **VELOCITY** | +0.31^NS^ | -0.13^NS^ | -0.31^NS^ | +0.38^NS^ | +0.08^NS^ | +0.32^NS^ | ― |

^**^P<0.01, ^*^P<0.05, ^NS^P>0.05.
